# Outcomes of MIS-C patients treated with anakinra: a retrospective multicenter national study

**DOI:** 10.3389/fped.2023.1137051

**Published:** 2023-08-22

**Authors:** Francesco Licciardi, Carlotta Covizzi, Marta Dellepiane, Nicole Olivini, Maria Vincenza Mastrolia, Andrea Lo Vecchio, Viviana Monno, Maria Tardi, Angela Mauro, Maria Alessio, Giovanni Filocamo, Marco Cattalini, Andrea Taddio, Roberta Caorsi, Gian Luigi Marseglia, Francesco La Torre, Andrea Campana, Gabriele Simonini, Angelo Ravelli, Davide Montin

**Affiliations:** ^1^ Division of Pediatric Immunology and Rheumatology, Department of Public Health and Pediatrics, “Regina Margherita” Children Hospital, University of Turin, Turin, Italy; ^2^ Pediatrics Unit, University Department of Pediatrics (DEAPG), Bambino Gesù Children’s Hospital—IRCCS, Rome, Italy; ^3^ Rheumatology Unit, Department of Paediatrics, Meyer Children’s Hospital, University of Florence, Florence, Italy; ^4^ Section of Paediatrics, Department of Translational Medical Sciences, University of Naples Federico II, Naples, Italy; ^5^ Pediatric Rheumatology Section, Department of Pediatrics, Giovanni XXIII Pediatric Hospital, Bari, Italy; ^6^ Rheumatology Unit, Department of Pediatrics, Santobono Pausilipon Children Hospital, Naples, Italy; ^7^ COVID Unit, Emergency Department, Santobono-Pausilipon Children Hospital, Naples, Italy; ^8^ Pediatric Rheumatology, Fondazione IRCCS Cà Granda Ospedale Maggiore Policlinico, Milano, Italy; ^9^ Spedali Civili, Unità di Immunologia e Reumatologia Pediatrica, Clinica Pediatrica dell’Università di Brescia, Brescia, Italy; ^10^ Institute for Maternal and Child Health “IRCCS Burlo Garofolo”, Trieste and University of Trieste, Trieste, Italy; ^11^ Clinica Pediatrica e Reumatologia, IRCCS Istituto Giannina Gaslini, Genoa, Italy; ^12^ Pediatric Clinic Foundation IRCCS Policlinico S. Matteo, University of Pavia, Pavia, Italy

**Keywords:** MIS-C, multisystem inflammatory syndrome, pediatrics, COVID-19, SARS-CoV2

## Abstract

**Background:**

The treatment of multisystem inflammatory syndrome in children unresponsive to first-line therapies (IVIG and/or steroids) is challenging. The effectiveness of IL-1 receptor antagonist, anakinra, is debated.

**Patients and methods:**

We conducted an anonymous retrospective multicenter study on MIS-C patients treated with anakinra in Italy from January 2020 to February 2021. Our study outcomes included the percentage of patients who required further therapeutic step-up, the percentage of patients who experienced fever resolution within 24 h and a reduction of CRP by half within 48 h, and the percentage of patients who developed Coronary Artery Anomalies (CAA) during follow-up.

**Results:**

35 cases of MIS-C were treated in 10 hospitals. Of these, 13 patients started anakinra while in the ICU, and 22 patients started anakinra in other wards. 25 patients (71.4%) were treated with corticosteroids at a starting dose 2–30 mg/Kg/day plus IVIG (2 g/Kg), 10 patients (28.6%) received only corticosteroids without IVIG. Anakinra was administered intravenously to all patients in Group A (mean dose 8 mg/Kg/day), and subcutaneously in Group B (mean dose 4 mg/Kg/day). Only two patients required further treatment step-up and no patients developed CAA after receiving anakinra. The most commonly observed side effect was an increase in ALT, occurring in 17.1% of patients.

**Conclusions:**

In this retrospective cohort of severe MIS-C patients treated with anakinra we report favorable clinical outcomes with a low incidence of side effects. The simultaneous use of steroids ± IVIG in these patients hinders definitive conclusions regarding the need of IL-1 inhibition in MIS-C treatment.

## Introduction

MIS-C is a severe complication of SARS-CoV2 infection in children, it often results in high rates of ICU admission, mainly due to acute cardiac insufficiency. Although most patients do not develop sequelae, in the acute phase MIS-C can be a life-threatening disease, making timely and appropriate treatment critical (1). Currently, the primary treatment options for MIS-C are IVIG and steroids. Numerous studies have established that MIS-C results from a cytokine storm involving various cytokines (2, 3). Therefore, biologic drugs, particularly the IL-1 receptor antagonist (anakinra), have shown promise in treating MIS-C, as evidenced by small case series. However, data on the overall efficacy and safety of anakinra in MIS-C treatment is still lacking (4–9).

## Methods

We conducted an anonymous retrospective multicenter study on patients who met the criteria for MIS-C and were treated with anakinra in Italy from January 4th, 2020 to February 28th, 2021. We included patients who met the WHO preliminary case definition (10) with recent biological evidence of SARS-CoV2 infection, either through serology or positive RT-PCR swab tests within six weeks prior to admission. The patients were divided into two groups: Group A included those who began anakinra treatment in the ICU, while Group B included patients treated in non-ICU pediatric wards. We collected clinical data at symptom onset, at the start of anakinra treatment, previous and concurrent treatments, along with reasons why anakinra was prescribed, dosage, and route of administration. The reasons for starting anakinra, according to the prescriber, included: worsening of cardiac dysfunction (defined as a reduction of EF to less than 50% or persistent hypotension requiring inotrope support), persistent clinical symptoms (persistence of fever with or without persistent mucocutaneous manifestations), or abnormal laboratory results (failure to reduce CRP or progressive increase of ferritin 24–36 h) despite treatment with steroid ± IVIG. We considered as outcomes the percentage of patients who required further therapeutic step-up, the percentage of patients with fever resolution within 24 h and CRP reduction by half within 48 h, and the percentage of patients with Coronary Artery Anomalies (CAA) during follow-up. We defined CAA based on the 2017 AHA Guidelines for Kawasaki Disease (11). The presence of CAA was screened at anakinra start, 2 weeks and 6–8 weeks after disease onset. In addition, we calculated the halving time of pro-BNP/BNP and CRP for each patient.

## Results

Overall, 35 cases of MIS-C were treated in 10 hospitals throughout Italy and met the inclusion criteria, with 13 in Group A and 22 in Group B. Table 1 displays baseline clinical and laboratory features, while Table 2 provides information on first-line therapies and dosing, timing and reasons for anakinra initiation, anakinra dose, and method of administration. 25 patients were treated with corticosteroids at a starting dose 2–30 mg/Kg/day plus IVIG (2 g/Kg), 10 patients received only corticosteroids without IVIG. In Group A, cardiac function deterioration was the most common reason for starting anakinra (76.9%), while in Group B, anakinra was mostly started due to persistent clinical symptoms that were unresponsive to IVIG and/or steroids (54.5%). Of the 13 cases in Group A, 11 were admitted to ICU and initiated anakinra within 48 h of hospitalization. In all these patients anakinra was started less then 36 h after the start of steroid and/or IVIG. Anakinra was administered intravenously to all patients in Group A, with a mean dose of 8 mg/Kg/day. Meanwhile, the majority of patients in Group B (72.7%) received the drug via subcutaneous route, with a mean dose of 4 mg/Kg/day. Table 3 outlines the outcomes and side effects experienced by these patients. In summary, only two patients required further treatment step-up: one patient in Group A needed an increase in methylprednisolone dose, while another patient in Group B required IVIG. No patients developed CAA after receiving anakinra. The most commonly observed side effect was an increase in ALT with 30.8% of Group A patients and 9.1% of Group B patients experiencing this effect. Only one patient in the study experienced an injection site reaction.

**Table 1:** Baseline clinical and laboratory features.

	Group A	Group B
N. of patients	13 (37.1%)	22 (62.9%)
Age at diagnosis (years)	12 (8–13)	8.5 (4–12)
Positive SARS-CoV2 swab in the six weeks previous to admission	84.6%	31.8%
IgG SARS-Cov2 positivity	100%	100%
Clinical presentation		
Days of fever	5 (4–6)	5 (4–6)
Conjunctivitis	53.8%	81.8%
Mucositis	23.1%	54.5%
Lymphadenopathy	23.1%	45.5%
Hands and feet erythema/oedema	7.7%	18.2%
Rash	46.2%	50.0%
Diarrhea	61.5%	50.0%
Abdominal pain	61.5%	86.4%
Hypotension^a^	61.5%	40.9%
Blood exams (at anakinra start)		
Platelets (/mmc3)	185 (107–189)	152 (89–187)
WBC (/mmc3)	10,780 (5,020–14,280)	10,935 (6,155–18,500)
Lymphocytes (/mmc3)	960 (660–980)	1,710 (760–1,970)
Neutrophil (/mmc3)	9,820 (4,280–13,310)	7,175 (4,370–17,020)
AST (UI/L)	40 (27–69)	31 (25–58)
ALT (UI/L)	33 (21–53)	40 (20–47)
Na (mmol/L)	133 (132–135)	137 (134–138)
Albumin (g/dl)	3.1 (2.7–3.4)	2.9 (2.4–3.1)
CRP (mg/L)	293 (212–305)	86 (17–153)
Procalcitonin (ng/ml)	11.3 (3.5–49.0)	1 (0.3–12.6)
Ferritin (ng/ml)	974 (419–4,006)	598 (447–1,366)
Cardio-pulmonary alterations (at anakinra start)		
Hypotension^a^	69.2%	36.4%
Need of inotrope support	69.2%	0%
Ejection fraction ≤50%	75.0%	23.8%
Pericardial effusion	44.4%	38.1%
BNP (ng/ml)	–	49 (7–123)
proBNP (ng/ml)	16,549 (3,247–20,163)	6,043 (1,405–10,502)
CAA	0%	9,1%
Pleural effusion	30.8%	4.5%
Need of O2 supplementation	61.5%	13.6%

All the continuous variables are expressed as median and IQR.

MV, mechanical ventilation; NIV, noninvasive ventilation; HFNC, high flow nasal cannula; NC, nasal cannula.

^a^ Below 5° percentile, according to age, gender, and height adjusted charts (Schwandt et al. Am J Hypertens. 2015).

**Table 2:** Reasons for anakinra start, anakinra dose and route of administration.

Reason for anakinra prescription		
Worsening of cardiac dysfunction^a^	76.9%	45.4%
Persistent clinical symptoms^a^	38.5%	54.5%
Persistent alteration of blood exams abnormalities^a^	46.2%	49.9%
Anakinra prescription		
Time between admission and anakinra start (days)	1 (1–2)	3 (1–7)
Iv administration	100%	27.3%
Sc administration	0%	72.7%
Mean dose (mg/kg/day)	8 (6.4–10)	4 (4–5.2)
Duration of anakinra treatment (days, with tapering)	35 (32–35)	27 (14–54)
First-line therapy		
Patients treated with IVIG (2 g/kg) without corticosteroids	0%	0%
Patients treated with IVIG (2 g/kg) + corticosteroids (2–30 mg/kg/day iv)	69.2%	72.7%
Patients treated with corticosteroids (2–30 mg/kg/day iv)	30.8%	27.3%

All the continuous variables are expressed as median and IQR.

^a^ Multiple choices were possible.

**Table 3:** Outcomes and side effects.

	Group A	Group B
N. of patients	13 (37.1%)	22 (62.9%)
Outcomes		
Need of treatment step-up after anakinra	7.7%	4.5%
Defervescence of fever at 24 h	100%	77.3%
CRP halving at 48 h	76.9%	72.7%
CAA development after anakinra	0%	0%
CRP halving time (days)	2 (1–2)	2 (1–3)
BNP/proBNP halving time (days)	2.5 (2–3)	2 (2–4)
Ejection Fraction normalization time (days)	2 (2–3)	3 (2–9)
Need of ICU admission after anakinra	–	0%
ICU admission duration after anakinra	5 (4–11)	–
Time to inotrope support halving (days)	2 (1–3)	–
MIS-C relapse after anakinra suspension	0%	0%
Side effects		
ALT increase during treatment (>2× age range)	30.8%	9.1%
Injection site reaction	–	4.5%

All the continuous variables are expressed as median and IQR.

## Discussion

MIS-C is a life-threatening late complication of SARS-COV2 infection in children. The treatment of MIS-C has been extensively debated over the past few years and is mainly based on the use of IVIG and/or glucocorticoids. According to most international recommendations, (12, 13) anakinra is suggested as a second-line therapy in patients resistant to conventional therapy. These recommendations are based mainly on individual case or small case series. A recent revision by Mastrolia et al. of these published cases found an efficacy of anakinra in 85/87 (97.7%) patients, nevertheless this result is burdened by publication bias and the outcome measures are extremely heterogenous (14). In this report, we describe the outcomes of a large multicenter cohort of MIS-C patients treated with anakinra. In both Italy and the US, anakinra is considered a second-line therapy for MIS-C (12, 13). Therefore, our retrospective cohort includes patients who either had very severe MIS-C at onset or were unresponsive to first-line therapy. Despite this selection bias, the majority of our patients achieved positive clinical and laboratory outcomes. Specifically, 85.7% of patients experienced fever remission within 24 h, while the median CRP and pro-BNP halving time was 2 days. Recently, Çaǧlayan et al. published a retrospective study of 82 MIS-C patients treated with anakinra. Of them, 89.1% were discharged without sequelae, but seven patients died (8.5%). In contrast, none of the patients in our study died. The lower mortality in our cohort may be due to several possible factors, such as a different ethnic background and a higher mean dose of anakinra (5.5 mg/Kg/day vs. 2.7 mg/Kg/day in Çaǧlayan et al.) (9). Our study confirms that anakinra may have a role as a second line treatment after glucocorticoids and/or IVIG. In Group B, only one patient (4.5%) required further therapeutic intervention after starting anakinra, none required admission to the ICU or vasopressor support. The majority experienced rapid fever resolution (77.3% at +24 h) and normalization of EF within an average of three days. It should be highlighted that 10 patients did not receive IVIG prior to or during anakinra treatment. Of these patients, 90% achieved fever remission within 24 h and halved their CRP levels within 48 h when treated with a combination of anakinra and intravenous methylprednisolone, only 1 patient required further therapeutic step-up with IVIG. This suggests that the anakinra and intravenous methylprednisolone combination may be effective even without IVIG. After the first revision of our manuscript, Chang et al. published a multicenter retrospective study analyzing the effect of anakinra therapy in the early phase of MIS-C treatment. The authors compared 121 MIS-C patients treated with anakinra plus IVIG and/or glucocorticoids to 389 propensity-score matched MIS-C patients treated without anakinra, and found that the treatment with anakinra was not associated with significant differences in vasopressor requirement, ventricular dysfunction, or C-reactive protein reduction (15). The results of this recently published paper are partially comparable with ours, in fact Chang et al. only included patients who received anakinra on days 0 or 1 after diagnosis, which matches with only 11 patients in our cohort who were treated early with anakinra in the ICU setting. When comparing this similar subgroup of patients our outcomes were overall better than Chang's. For instance, the mean time to EF recovery was considerably shorter in our cohort (2 vs. 3 days). A possible explanation for this different outcome could be the higher mean dose of anakinra in our cohort (8 vs. 4 mg/Kg/day). It is worth mentioning that anti-IL1Ra autoantibodies have been described in small groups of children with MIS-C, and although their role in MIS-C pathogenesis is still debated, they can reduce free IL1-Ra, thereby possibly antagonizing anakinra and increasing the need for anakinra in MIS-C treatment (16). In accordance with this hypothesis, previous papers report the efficacy of high doses of anakinra in MIS-C (up to 10 mg/Kg/day) (5, 17). With regards to vasopressor support, the outcome considered in the aforementioned paper (% of patients needing vasopressor support at day 3) might not have been sensitive enough to detect the benefit of anakinra. In fact, in our experience, anakinra had a benefit in terms of vasopressor tapering (mean time for halving vasopressor dose was 48 h), but not in terms of early suspension. Finally, it should be highlighted that the incidence of CAA in MIS-C worldwide is between 10%–20%. In 2020, an Italian multicenter survey reported an incidence of 13.2% (12, 18). Interestingly, none of the patients in our cohort developed CAA after receiving anakinra, possibly indicating a protective role of IL-1 inhibition on coronary arteries during the acute phase of the disease, similar to that observed in KD (19).

This study has some limitations. Firstly, the data were collected retrospectively and there was no shared protocol between centers on when and how starting anakinra. The lack of a protocol led to a heterogeneity in timing and dosing between different hospitals. Additionally, the absence of a control group hinders the ability to draw a firm conclusion regarding the benefits of anakinra, particularly at disease onset when anti-IL1 is initiated shortly after steroid use.

In summary, in this retrospective cohort of patients treated with anakinra, we have observed favorable clinical outcomes with a low incidence of side effects, mainly transient ALT elevation. However, it is crucial to note that anakinra was administered in combination with other therapies, primarily steroids, which makes it difficult to draw definitive conclusions about the role of IL-1 inhibition in MIS-C treatment. Our data is not conclusive, especially in the ICU setting where anakinra was used simultaneously with IVIG and steroids.

Prospective randomized trials should be conducted to confirm our findings and determine the optimal timing and dose of anakinra for MIS-C treatment. Additionally, the development of a first-line therapies resistance score, similar to the Kobayashi score for IVIG resistance in Kawasaki Disease, might help identify which patients might benefit from prompt IL-1 inhibition.

## Data Availability

The original contributions presented in the study are included in the article/Supplementary Material, further inquiries can be directed to the corresponding author.
